# Interleukin-1 as a mediator of fatigue in disease: a narrative review

**DOI:** 10.1186/s12974-017-0796-7

**Published:** 2017-01-21

**Authors:** Megan E. Roerink, Marieke E. van der Schaaf, Charles A. Dinarello, Hans Knoop, Jos W. M. van der Meer

**Affiliations:** 1 Department of Internal Medicine, Radboud University Medical Centre, Geert Grooteplein Zuid 8, 6500HB Nijmegen, The Netherlands; 2 Expert Centre for Chronic Fatigue, Radboud University Medical Centre, Reinier Postlaan 4, 6525GC Nijmegen, The Netherlands; 3 Department of Medicine, University of Colorado Denver, 12700 E. 19th Avenue Box B168, Aurora, CO 80045 USA; 4 Department of Medical Psychology, Academic Medical Centre (AMC), University of Amsterdam, Meibergdreef 9, 1105AZ Amsterdam, The Netherlands

**Keywords:** Fatigue, Interleukin 1, Inhibition, Treatment

## Abstract

Fatigue is commonly reported in a variety of illnesses, and it has major impact on quality of life. Previously, it was thought that fatigue originates in the skeletal muscles, leading to cessation of activity. However, more recently, it has become clear that the brain is the central regulator of fatigue perception. It has been suggested that pro-inflammatory cytokines, especially interleukin-1 alpha (IL-1α) and interleukin-1 beta (IL-1β), play a prominent role in the development of central fatigue, and several studies have been performed to elucidate the connection between inflammation and these central processes.

In this narrative review, mechanisms of action of IL-1 are described, with special attention to its effect on the central nervous system. In addition, we present a summary of studies that (i) investigated the relationship between circulating IL-1α and IL-1β and fatigue severity and/or (ii) evaluated the effect of inhibiting IL-1 on fatigue. We aim to improve the understanding of fatigue in both inflammatory and non-inflammatory illnesses, which could help develop strategies to treat fatigue more effectively.

Reviewing the studies that have been performed, it appears that there is a limited value of measuring circulating IL-1. However, inhibiting IL-1 has a positive effect on severe fatigue in most studies that have been conducted.

## Background

### General introduction and aims

There is growing evidence supporting the theory that the central nervous system plays an important role in the perception of fatigue. The central nervous system processes and values sensory information, as well as guides motivational behavior involving decisions to discontinue activity or to invest effort. Cytokines have been suggested as prominent mediators in the induction of this central fatigue.

In this narrative review, we explored the evidence for the connection between pro-inflammatory cytokines, especially interleukin-1 (IL-1), and the perception of fatigue. Next to investigations that have examined whether there is a relation between circulating IL-1 and severity of fatigue (Table 1), the effect of blocking IL-1 on fatigue severity has also been reported (Table 2). For example, trials have been performed in rheumatoid arthritis [1, 2], Sjögren’s syndrome [3], and diabetes [4]. In this review, the different mechanisms of action of IL-1 will be discussed, especially considering its action in the CNS. We also review studies performed up to this writing that searched for a relation between IL-1 and fatigue in a variety of inflammatory and non-inflammatory illnesses.

**Table 1:** Overview of studies measuring IL-1 in patients reporting fatigue

Reference	Disease activity	Number of patients	Fatigue questionnaire	IL-1 measurement	Main outcome
Rheumatoid arthritis					
Lampa et al., 2014	No neurological disease or generalized pain nor of swollen joints 4.9 ± 3.8	Patients (*n* = 14), controls (*n* = 12)	VAS-fatigue	CSF IL-1 and IL-1Ra	Higher IL-1β and lower IL-Ra in RA vs controls (*p* < 0.001, *p* < 0.05), positive correlation between IL-1β and fatigue (*R* = 0.55, *p* < 0.05)
Sjögrens syndrome					
Harboe et al., 2009	No acute illness in the week prior to or after sampling, no CRP/ESR elevation	Patients (*n* = 54), controls (*n* = 53)	FSS, VAS-fatigue	CSF IL-1β, IL-1Ra, and IL-1sRII	Higher IL-1Ra in patients (*p* = 0.026), correlation IL-1Ra and VAS-fatigue (*R* ^2^ = 0.11, *p* = 0.015).
Sarcoidosis					
Korenromp et al., 2011	No disease activity	Fatigued patients (*n* = 34), non-fatigued patients (*n* = 38)	CIS-fatigue (severe fatigue when ≥35)	Plasma IL-1α, IL-1β, and IL-1Ra	No significant differences
				Whole blood production of IL-1α and IL-1β after stimulation with LPS (1 ng/ml)	
Baydur et al., 2010	Pulmonary sarcoidosis	Patients (*n* = 22), controls (*n* = 22)	MFI-20	Plasma IL-1β before, directly after and 4–6 h after exercise	Higher fatigue scores in sarcoidosis patients (*p* < 0.0001). IL-1β not different between patients and controls or among the three collection times. Correlation between pre-exercise IL-1β and MFI-20 in patients receiving immunomodulatory medication (*R* ^2^ 0.63, *p* = 0.03).
Cancer					
Mixed (cancer survivors plus advanced cancer)					
De Raaf et al., 2012	Advanced cancer or 1–5 years post cancer treatment	Advanced cancer (*n* = 45), cancer survivors (*n* = 47)	MFI	Plasma IL-1Ra	Advanced cancer patients had higher IL-1Ra concentrations (*p* < 0.01). In these patients, physical fatigue was correlated with IL-1Ra (*r* = 0.32, *p* = 0.03). In cancer survivors, IL-1Ra was related to both physical (*r* = 0.24, *p* = 0.10) and mental fatigue (*r* = 0.35, *p* = 0.02).
Prostate cancer					
Greenberg et al.,1993	Men undergoing localized radiotherapy	Patients (*n* = 15)	VAS-fatigue/daily during 8 weeks	Serum IL-1β, at baseline and weekly thereafter	A rise in fatigue was seen between weeks 1 and 4, fatigue stabilized during week 5 and increased again in weeks 6 and 7. Rise in fatigue during the first 4 weeks was accompanied by increased IL-1β concentrations (*p*-value not reported).
Bower et al., 2009	Patients undergoing external beam radiation therapy	Prostate cancer (*n* = 20), breast cancer (*n* = 28)	FSI/fatigue during the past week/baseline, after 5/10/20 days of treatment, final week of treatment, and 2 weeks and 2 months after treatment	Serum IL-1β, IL-1Ra in a subset of patients, at same time-points as the questionnaires	Fatigue increased in both groups during treatment. Significant quadratic trend for IL-1β during treatment (*p* = 0.034). Treatment dose was not associated with IL-1β and IL-1Ra concentrations. There was no correlation between IL-1β and fatigue severity. IL-1Ra was associated with fatigue (*β* = 0.63, *p* = 0.016).
Dirksen et al., 2014	Non-metastatic cancer prior to radiation therapy	Patients (*n* = 30)	POMS fatigue (inertia subscale)/pre-treatment en post-treatment	Serum IL-1β, pre-treatment and post-treatment (<2 weeks after radiotherapy, <10 weeks after brachytherapy)	Fatigue was increased post-treatment (*p* = 0.027). No differences in IL-1β concentrations, no correlation with fatigue severity
Jim et al., 2012	Non-metastatic or asymptomatic metastatic prostate cancer	Patients (*n* = 53)	FSI (fatigue over the past week)/at baseline and after 6 months	SNP in IL1B gene (rs16944)	IL1B had no significant effect on fatigue-related outcomes
Breast cancer					
Geinitz et al., 2000	Women undergoing postoperative radiotherapy (no chemotherapy), without metastatic disease	Patients (*n* = 41)	FAQ, and VAS-fatigue/during previous week/at baseline, end of weeks 1–5, and 2 months after treatment	Serum IL-1β, same time points as questionnaires	VAS-fatigue increased until week 4 (*p* < 0.001). During weeks 4 and 5 FAQ physical (*p* = 0.035 and 0.015) and cognitive (*p* = 0.008 and 0.007) subscales were significantly elevated. IL-1β did not increase during treatment.
Von Ah et al., 2008	Stage 0–IIIa breast cancer before adjuvant therapy	Patients (*n* = 44)	Piper-fatigue scale/at baseline and at 3 months (during adjuvant therapy) and 6 months after baseline (initial recovery)	Whole blood production of IL-1β after stimulation with PHA (10 μg/ml)	IL-1β predicted fatigue before adjuvant therapy (*β* = 0.30, *p* < 0.05).
Liu et al., 2012	Stage I–III breast cancer prior to ≥4 3-week cycles of chemotherapy	Patients (*n* = 53)	MFSI-SF/fatigue during past week/at baseline and during cycles 1 and 4 of chemotherapy (last 2 weeks)	Plasma IL-1Ra, at the same time points as questionnaires	Fatigue significantly increased over time (*p* < 0.05). IL-1Ra dropped at cycle 1 week 3 (*p* < 0.0001). There was no association between IL-1Ra and fatigue.
Schmidt et al., 2015	Stage 0–III breast cancer prior to adjuvant radiation therapy	Patients (*n* = 92)	FAQ/at baseline, after completion of radiotherapy (week 7), and the end of the intervention (week 13, resistant exercise/relaxation)	Serum IL-1Ra, at the same time points as questionnaires	Moderate correlation between IL-6/IL-1Ra at the end of radiotherapy with physical fatigue at the same time (*r* = 0.25, *p* = 0.022) and at 6 weeks after chemotherapy (*r* = 0.23, *p* = 0.046).
Bower et al., 2002	Stage 0–II breast cancer 1–5 years after diagnosis, after completion of treatment	Fatigued (*n* = 20), non-fatigued (*n* = 20)	Energy/fatigue subscale RAND-36 (score 0–50 = high fatigue, score 70–100 = low fatigue)/fatigue during past 4 weeks FSI/fatigue during past week	Serum IL-1β and IL-1Ra	Fatigued women had significantly higher IL-1Ra concentrations (*p* = 0.006).
Bower et al., 2011	Stage 0–IIIA breast cancer, after completion of primary cancer therapy (within past 3 months) i.e., surgery, radiation, and/or chemotherapy	Patients (*n* = 103)	FSI (cut-off 3)/fatigue during the past week	Plasma IL-1Ra	64% scored above 3 on the FSI; these patients did not have a higher IL-1Ra concentration. There was no significant association between IL-1Ra and fatigue or chemotherapy exposure.
Bower et al., 2007	Stage 0–II breast cancer survivors (6.5–10 years after diagnosis)	Fatigued (*n* = 10), non-fatigued (*n* = 15)	Vitality scale SF-36 (<50 = significant fatigue, >70 = absence of significant fatigue)	Whole blood production of IL-1β after stimulation with LPS (100 pg/ml) or cortisol (0, 10^−8^, 10^−^7, 10^−6^M), at baseline, directly after TSST, and after 30 min recovery	No differences at baseline. IL-1β increased significantly in fatigued patients after completion of the TSST (*p* = 0.02).
Collado-Hidalgo et al., 2006	Stage 0–III breast cancer survivors, 1–5 years post-diagnosis	Fatigued (*n* = 32), non-fatigued (*n* = 18)	Vitality scale SF-36 (<50 = significant fatigue, >70 = absence of significant fatigue)	Plasma IL-1Ra	IL-1Ra was significantly higher in fatigued breast-cancer survivors (*p* = 0.05).
Orre et al., 2011	Stage II–III breast cancer patients, 2.7–7.2 years after postoperative locoregional radiotherapy	Patients (*n* = 299)	Fatigue questionnaire	Serum IL-1Ra	There was no significant association between IL-1Ra and fatigue.
Collado-Hidalgo et al., 2009	Stage 0–III breast cancer survivors, 1–5 years post-diagnosis	Fatigued (*n* = 33), non-fatigued (*n* = 14)	Vitality scale SF-36 (≤55 = significant fatigue, >70 = absence of significant fatigue), MFSI	IL-1B-511 (CT) polymorphism	Fatigued survivors had a substantial overrepresentation of CC alleles, and underrepresentation of TT alleles. The prevalence of at least one cytosine was more frequent among fatigued patients (*p* = 0.007) and associated with fatigue in multiple regression (*p* = 0.021). Which was no longer significant after controlling for depressive symptoms (*p* = 0.052).
Reinertsen et al., 2011	Stage II–III breast cancer survivors	Fatigued (*n* = 101), non-fatigued (*n* = 201)	Fatigue questionnaire (cut-off 4, clinical significant fatigue), chronic fatigue was defined as fatigue being present for at least 6 months	IL-1B rs16944 (A/G) SNP, and IL-1β mRNA expression	There was no association between chronic fatigue and the IL-1B SNP or IL-1β mRNA expression.
Testicular cancer					
Orre et al., 2009	Patients 5–20 years after unilateral orchiectomy	Fatigued (*n* = 92), non-fatigued (*n* = 191)	Fatigue questionnaire (cut-off 4, clinical significant fatigue), chronic fatigue was defined as fatigue being present for at least 6 months	Plasma IL-1Ra	Fatigued patients had significant higher IL-1Ra (*p* = 0.002). In multiple regression analysis, IL-1Ra corrected for age had an OR of 1.93 (95%CI 1.21–3.08). Although age an IL-1Ra explained only 4% of the variance. IL-1Ra was not included in the final model.
Uterine cancer					
Ahlberg et al., 2004	Patients receiving external radiation therapy after hysterectomy	Patients (*n* = 15)	MFI-20/at baseline, after 30Gy (+3 weeks) and after 46Gy (+5–6 weeks)	Plasma IL-1 (α or β unknown), same time-points as questionnaires	Fatigue increased during treatment, IL-1 remained below the detection limit during the entire study period (4 pg/ml).
AML/MDS					
Meyers et al., 2005	Newly diagnosed AML/MDS before undergoing chemotherapy.	Patients (*n* = 54)	Brief fatigue inventory (cut-off score ≥4, moderate-severe fatigue)/fatigue in the past 24 h/baseline and after 1 month of treatment	Plasma IL-1 (α or β unknown) and IL-1Ra, at baseline.	There was a positive correlation of IL-1Ra and fatigue (*r* = 0.52, *p* value not reported).
Post-stroke fatigue					
Ormstad et al., 2011	Acute stroke patients	Patients (*n* = 45)	FSS (dichotomized as a score ≥4 or <4)/at 6, 12, and 18 months after stroke	Serum IL-1β and IL-1Ra, <24h (*n* = 35), 24–48 h (*n* = 7), and 48–72 h (*n* = 3) after stroke onset	Significant correlation between IL-1β and fatigue at 6 months (*r* = 0.37, *p* = 0.015). Negative correlation between IL-1Ra and fatigue at 12 months (*r* = −0.38, *p* = 0.013). Fatigued patients had significant lower IL-1Ra concentrations.
Becker et al., 2015	Acute stroke patients	Patients (*n* = 39)	FAS/30/90/180/365 days after stroke	IL1RN SNP rs4251961	Carriers of a C allele reported more fatigue (*p* = 0.03). At 30 and 90 days, patients with at least one C allele had higher scores on fatigue (*p* < 0.05).
CFS					
Hornig et al., 2015	CFS	Patients (illness duration ≤3 years *n* = 52, illness duration >3 years *n* = 246), controls (*n* = 348)	MFI	Plasma IL-1α, IL-1β and IL-1Ra	There were no differences when comparing all patients combined to controls. However, patients with a short illness duration had significantly higher IL-1α (*p* < 0.05) and IL-1Ra (*p* < 0.05) compared to controls. In patients with a long illness duration, IL-1β was significantly lower compared to controls (*p* < 0.05). IL-1α, IL-1β and IL-1Ra were higher in short illness patients compared to long illness patients (*p* < 0.01).
Russell et al., 2016	CFS (female)	Patients; 1. ≤18/illness duration ≤2 years (*n* = 18), 2. age 18–50/average illness duration 7 years (*n* = 22), 3. age ≥50 and average illness duration 11 years (*n* = 28), controls (*n* = 81)	Chalder fatigue in adolescents, and MFI in other patients	Plasma IL-1α and IL-1β	Looking at individual expression, there were no differences between patients and controls. IL-1α appeared in a linear classification model in the adolescent group, but not in the other 2 groups.
Hardcastle et al., 2015	Moderate (mobile) or severe (housebound) CFS	Moderate CFS (*n* = 22), severe CFS (*n* = 19), controls (*n* = 22)	FSS	Serum IL-1β and IL-1Ra	Significant IL-1β increase in moderate compared with severe CFS patients (*p* = 0.002). For other subgroups and IL-1Ra there were no differences.
Landi et al., 2016	CFS	Patients (*n* = 100), controls (*n* = 79)	MFI	Plasma IL-1α and IL-1β	No significant differences.
Chao et al., 1991	CFS	Patients (*n* = 9), controls (*n* = 7)	VAS-fatigue	Serum IL-1β	No differences in serum IL-1β. IL-1β production after LPS stimulation was significantly higher in CFS patients (*p* < 0.05)
				PBMC production of IL-1β after stimulation with LPS (1 ng/ml) or PHA (4 μg/ml)	
Swanink et al., 1996	CFS	Patients (*n* = 76), controls (*n* = 69)	CIS	Plasma IL-1α, IL-1β, and IL-1Ra	No differences in circulating cytokine concentrations. Significant lower IL-1β production after LPS stimulation (*p* < 0.05), no correlation between production and fatigue severity.
				Whole blood production of IL-1α, IL-1β, and IL-1Ra after stimulation with LPS	
Mawle et al., 1997	CFS	Patients (*n* = 26), controls (*n* = 50)	–	PBL production of IL-1α and IL-1β after stimulation with PHA	IL-1α production was lower in severely ill patients (*n* = 13) and those with a gradual disease onset (*n* = 17) compared to controls (*p* = 0.038, *p* = 0.011). IL-1β was also lower in patients with a gradual disease onset (*p* = 0.039).
Cannon et al., 1997	Sudden onset CFS	Patients (*n* = 16), controls (*n* = 15)	–	PBMC production of IL-1β, IL-1Ra, and IL-1sRII after stimulation with LPS (1 ng/ml), indomethacin, or a combination, before and daily after a 15 min exercise on day 2	At baseline, controls had a significant increase in IL-1β production during the luteal phase (unstimulated, *p* = 0.021). This increase was absent in CFS patients. In the follicular phase, control group had an increase IL-1β production 48 h after exercise. In CFS patients, there was no alteration over time. In the follicular phase, IL-1Ra secretion was higher in CFS patients (unstimulated, *p* = 0.023). IL-1sRII was higher in patients (unstimulated, *p* = 0.0002).
Tomoda et al., 2005	CFS	Patients (*n* = 15), controls (*n* = 23)	–	IL-1β production of PBMCs after stimulation with PHA (5 μg/ml) or LPS (50 ng/ml)	No significant differences.
Lloyd et al., 1991	CFS	Patients (*n* = 25), controls (*n* = 28)	–	Serum and CSF IL-1β	No significant differences.
Peterson et al., 2015	CFS	Patients (*n* = 18), controls (*n* = 5)	–	CSF IL-1β and IL-1Ra	No significant differences.
Natelson et al., 2005	CFS	Patients (*n* = 44), controls (*n* = 13)	MFI	CSF IL-1α and IL-1β	No significant differences.
Hornig et al., 2016	CFS	Patients (*n* = 32), MS controls (*n* = 40), and controls (*n* = 19)	–	CSF IL-1α, IL-1β and IL-1Ra	CFS patients had significant lower IL-1β and IL-1Ra concentrations compared to normal controls (*p* = 0.003 and *p* = 0.014). And compared to MS patients IL-1α (*p* = 0.0007), IL-1β (*p* = 0.0018) and IL-1Ra (*p* = 0.0003) were decreased in CFS.
(Post-)infectious fatigue					
Vollmer-Conna et al., 2004	Patients with acute Q-fever, EBV, or RRV	Q-fever (*n* = 18), EBV (*n* = 24), RRV (*n* = 24)	Physical symptom checklist/fatigue in the past 2 weeks	Serum IL-1β	Fatigue was reported in 100% of Q-fever patients, >75% of EBV patients, and >50% of RRV patients. In Q-fever, IL-1β correlated significantly with fatigue (*r* = 0.47, *p* = 0.04), which was also found in the EBV/RRV combination group (*r* = 0.39, *p* = 0.01). All significant results were obtained from the unstimulated samples.
				PBMC production of IL-1β after stimulation with LPS (10 ng/ml)	
Vollmer-Conna et al., 2007	Patients with post-infectious fatigue and post-infectious patients without fatigue	EBV (*n* = 11), RRV (*n* = 6), Q-fever (*n* = 5), and controls after EBV (*n* = 17), RRV (*n* = 14) or Q-fever (*n* = 11)	Somatic and psychological health report (fatigue was defined as a score ≥3 on the SOMA subscale)/at 1, 2, 3, 6, and 12 months after onset of the infection	Serum IL-1β	No significant differences.
				PBMC production of IL-1β after stimulation with LPS (10 ng/ml), mouse anti-human or anti-CD3	

An overview of all studies that investigated the relationship between IL-1 and fatigue severity

*Abbreviations*: *AML* acute myeloid leukemia, *CFS* chronic fatigue syndrome, *CSF* cerebrospinal fluid, *CIS* checklist individual strength, *CRP* C-reactive protein, *EBV* Epstein-Barr virus, *ESR* erythrocyte sedimentation rate, *FAS* fatigue assessment scale, *FAQ* functional activity questionnaire, *FSI* fatigue symptom inventory, *FSS* fatigue severity scale, *LPS* lipopolysaccharide, *MDS* myelodysplastic syndrome, *MFI* multidimensional fatigue inventory, *MFSI* multidimensional fatigue symptom inventory, *MS* multiple sclerosis, *PBL* peripheral blood leukocytes, *PBMC* peripheral blood mononuclear cell, *PHA* phytohaemagglutinin, *POMS* profile of mood states, *RRV* Ross river virus, *SF* short form, *TSST* Trier social stress test, *VAS* visual analog scale

**Table 2:** Overview of studies evaluating the effect of inhibiting IL-1 on fatigue severity

Reference	Disease activity	Design	Number of patients	Fatigue questionnaire	IL-1 intervention	Main outcome
Rheumatoid arthritis						
Alten et al., 2011	≥6 of 28 tender and swollen joints, elevated hsCRP and/or ESR	Randomized, double-blind, placebo-controlled, parallel-group, dose-finding trial	274	FACIT-F at 12 weeks	MTX combined with canakinumab: (1.) 150mg s.c. every 4 weeks (*n* = 69), (2.) 300 mg s.c. every 2 weeks (*n* = 64), (3.) 600 mg i.v. followed by 300 mg s.c. every 2 weeks (*n* = 71) or placebo s.c. every 2 weeks (*n* = 70)	Decrease in fatigue canakinumab group 1 (*p* = 0.006) and 3 (*p* = 0.028) compared to placebo.
Omdal et al., 2005	Mean DAS28 6.2 ± 1.1	Pilot, non-blinded, no control group	8	FSS and VAS-fatigue at baseline, week 4, and week 8	100 mg s.c. anakinra daily	Decrease in FSS (*p* = 0.002) and VAS-fatigue (*p* = 0.0001) during the 8 weeks, accompanied by a decrease of the DAS28 score (*p* < 0.0001).
Sjögrens syndrome						
Norheim et al., 2012	No elevation CRP/ESR	Randomized, double-blind, placebo-controlled, parallel-group trial	26, 1 not included in analysis	FSS and VAS-fatigue at baseline, week 0, week 2, week 4, and week 5	100 mg s.c. anakinra (*n* = 12) or placebo (*n* = 13) daily during 4 weeks	No difference FSS scores after 4 weeks, more frequent reduction of VAS-fatigue of >50% in anakinra group (50 vs 8%, *p* = 0.03).
CAPS						
Kone-Paut et al., 2011	Moderate or severe disease activity	Part 1. open-label, followed by part 2. which was a double-blind withdrawal phase in responders, ending with open-label part 3	35	5-point likert scale, daily first 15 days of part 1, weekly thereafter (physician and patient), FACIT-F	Single canakinumab (150 mg) dose in part 1 (*n* = 35), followed by canakinumab (*n* = 15) or placebo (*n* = 16) every 8 weeks for 24 weeks in part 2. At relapse or at end of part 2, patients were treated with canakinumab for 16 more weeks (*n* = 31).	Fatigue absent or minimal at the end of part 1 in >85% of patients paralleled by decreased disease activity. Increase FACIT-F at the end of part 1 (*p* < 0.05). Fatigue relapse in patients randomized to placebo in part 2.
Huemmerle- Deschner, 2011	Disease activity requiring medical intervention	Open label, phase II trial	7 (pediatric)	5-point likert scale at post-treatment days 1 and 2, and weeks 1 and 5 (physician)	Canakinumab 150 mg or 2 mg/kg, repeated after 7 days in absence of complete response.	Fatigue was absent or minimal 1 day after canakinumab in all patients. This was accompanied by a decrease in disease activity.
Hoffman et al., 2008	NLPRP3 mutation combined with classic FCAS/MWS symptoms	Part 1. 6-week randomized controlled trial, part 2A. open-label, 2B. randomized controlled trial	47	DHAF rating fatigue over previous 24 h	Part 1 loading dose of 320 mg rilonacept/placebo s.c. (*n* = 47), followed by weekly s.c. injections of 160 mg rilonacept/placebo. Part 2 (*n* = 46) weekly s.c. rilonacept 160 mg during 9 weeks followed by 9 weeks rilonacept/placebo.	Decrease in fatigue in part 1 (*p* < 0.001), relapse in those patients treated with placebo in part 2 (*p* < 0.001).
Diabetes						
Cavelti-Weder et al., 2011	Type 2 diabetes	Randomized, double-blind, placebo-controlled trial	30	Fatigue scale for motor and cognitive functions	XOMA052/placebo (0.01–1 mg/kg)	At baseline, 53% of patients experienced mild-severe fatigue. One month after treatment, fatigue was increased in the placebo and lowest dosing group; in the two medium dosing groups, fatigue was slightly decreased; and in the two highest dosing groups, fatigue was remarkably decreased. Effect size dose-dependent effect *d* = 0.3. The highest dose of 1.0 mg/kg had a favorable effect on motor fatigue (*d* = 1.05).
Cancer						
Hong et al., 2015 [108]	Advanced non-small cell lung cancer	Open label dose escalation trial	16	EORTC-QLQ, at baseline and after 8 weeks	Intravenous MABp1 every 3 weeks trough 4 dosing levels (0.25/0.75/1.25/3.75 mg/kg, and 3.75 mg/kg every 2 weeks (until disease progression))	Non significant improvement in fatigue severity. Median disease free progression was 57 days.
Hickish et al., 2016	Metastatic colorectal cancer refractory to standard chemotherapy	Randomized controlled trial	309	EORTC-QLQ	MABp1 plus best supportive care or placebo (2:1)	Significant improvement of fatigue, increase in appetite, and decrease in pain severity

An overview of all studies that investigated the relationship between IL-1 and fatigue severity

*Abbreviations*: *CAPS* cryopyrin-associated periodic syndrome, *CRP* C-reactive protein, *DAS* disease activity score, *DHAF* daily health assessment form, *EORTC-QLQ* European organization for research and treatment of cancer quality of life questionnaire, *ESR* erythrocyte sedimentation rate, *FACIT-F* functional assessment of chronic illness therapy subscale fatigue, *FCAS* familial cold autoinflammatory syndrome, *FSS* fatigue severity scale, *hsCRP* high-sensitive C-reactive protein, *MTX* methotrexate, *MWS* Muckle-Wells syndrome, *s.c.* subcutaneous, *VAS* visual analog scale

### Interleukin-1

To elucidate the contribution of IL-1 to the experience of fatigue, it is important to have a view of the pleiotropic action of this cytokine. Because of the important role of IL-1 in the innate immune system and other physiological systems, it has become a field of great interest. Of the 11 members of the IL-1 family, two prominent members, IL-1alpha (IL-1α) and IL-1beta (IL-1β), have been described most frequently in the literature on fatigue. IL-1α, IL-1β, and the IL-1 receptor antagonist (IL-1Ra) bind to the type 1 IL-1 receptor (IL-1R1). Whereas IL-1α and IL-1β activate an inflammatory signal upon binding to the IL-1R1, IL-1Ra binds to the same receptor but does not activate a signal.

IL-1α is constitutively present as a bioactive precursor inside a wide range of cells. It is present, for example, in epithelial cells of the lungs, keratinocytes of the skin, and vascular endothelial cells [5]. During necrosis resulting in cell death, the bioactive IL-1α precursor is released. Furthermore, IL-1α is also present on the surface of monocytes and B lymphocytes [6]. IL-1β is produced by more specific subsets of cells; it is a product of monocytes, tissue macrophages, and dendritic cells [5]. In order to become biologically active, the IL-1β precursor is first cleaved by caspase-1, an intracellular enzyme that is activated by a complex of intracellular proteins termed “the inflammasome” [7]. There is also an alternative mechanism by which the inactive IL-1β precursor is converted into an active cytokine. In presence of a high numbers of neutrophils, enzymes released by these cells, such as elastase and proteinase-3, will cleave the IL-1β precursor and yield the bioactive moiety [8]. After binding of IL-1α or IL-1β to the IL-1R1, a complex signaling cascade is activated, eventually leading to “nuclear factor kappa-light-chain-enhancer of activated B cells” (NFκB) production and subsequent gene transcription [9]. In this manner, IL-1 action leads to a variety of biological events, ranging from activation of the acquired immune system to the induction of fever and slow-wave sleep [10]. For the scope of this review, we will focus on the ability of IL-1 to induce fatigue.

The importance when investigating the involvement of IL-1 in disease is to note that circulating concentrations of IL-1β often are at best only slightly elevated (picograms/ml) even under conditions of severe pathology [11]. A large part of IL-1β remains inside the cell, and in the circulation, it is bound to other proteins, such as the type 2 IL-1 receptor (IL-1R2), which serves as a decoy receptor, leading to a decrease in bioactivity [12]. Therefore, IL-1Ra, which is secreted by various cells in an inflammatory environment, has been proposed as a surrogate marker for IL-1β activity [12, 13].

### Effect of interleukin-1 on the central nervous system

The central nervous system (CNS) plays an important role in cytokine-induced fatigue. As stated earlier, IL-1α and IL-1β are produced by a broad range of immunocompetent and non-immunological cells. Elevation of IL-1 in the brain contributes to behavioral alterations described as “sickness behavior,” which includes increased feelings of fatigue and depressed mood, loss of interest in social interactions, and reduction of physical activity both in animals and in humans treated for different malignancies [14–19]. The observed behavioral alterations in response to the intrathecal administration of pro-inflammatory cytokines indicate that, in addition to its peripheral effect on the immune response, IL-1 also signals to the brain via several immune-to-brain communication pathways.

**Fig. 1:**
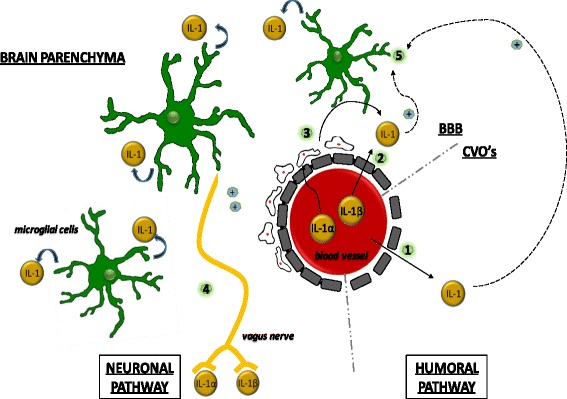
Overview of routes by which peripherally produced IL-1 is able to influence IL-1 levels in the brain. An overview of the five different routes that can be used by peripherally produced IL-1α and IL-1β to access the CNS. The first route (*1*) is diffusion of IL-1 trough the fenestrated endothelium surrounding blood vessels in the circumventricular organs (*CVOs*). The rest of the brain microvasculature is surrounded by the blood-brain barrier (BBB), where diffusion is not possible due to tight junctions between cells. In these areas, IL-1 can be transported across the BBB by a saturable transport system (*2*), or it can activate perivascular macrophages at the brain side of blood vessels, stimulating them to produce IL-1 (*3*). These three routes combined are frequently described as the humoral pathway, which is able to activate microglial cells in the brain parenchyma (*5*). Another important system is the neuronal pathway, where peripherally produced IL-1 stimulates afferent nerves, especially the vagal nerve, causing local IL-1 production in the CNS by microglial cells (*4*). Increased concentrations of IL-1 in different areas of the brain are suspected to influence neurotransmitter systems (e.g., dopamine and serotonin), thereby exerting its effect on behavior and the development of fatigue

Before peripherally produced cytokines can have an effect on the brain, they have to find a way to reach the CNS. In most diseases described in this review, there is no disruption of the blood-brain barrier (BBB) to allow proteins to gain access to the CNS. However, there are several mechanisms by which this barrier can be bypassed (Fig. 1). Some parts of the BBB are more permeable, especially those surrounding the circumventricular organs (CVOs), and cytokines like IL-1 can cross the BBB in this area by diffusion through the fenestrated endothelium (1) [20–22]. For IL-1α, IL-1β, and IL-1Ra, there is a saturable transport system from blood to the CNS (2) [20], and production of cytokines by locally activated perivascular endothelial cell and macrophages has also been described (3) [23]. These three routes combined are often described as the humoral pathway. There is also a neuronal pathway, which uses the vagal nerve and sometimes also other peripheral afferent nerve fibers (4), directly transmitting the cytokine signal to relevant brain regions [24]. The fifth route, activated by both the humoral and neuronal pathways, is activation of the immunocompetent cells of the brain, being the microglia (5). These cells are able to produce IL-1β locally once they have become activated [25, 26]. In chronic fatigue syndrome (CFS), a syndrome characterized by severe fatigue, evidence for microglial activation has already been reported in a small group of patients [27].

The IL-1R1 is distributed throughout the brain, although human studies on this topic are scarce [28]. The intracellular pathways after IL-1R1 activation in the brain are similar to those in the periphery, eventually leading to NFκB activation and subsequent gene transcription [28]. In an animal experiment, an increase of IL-1β messenger RNA (mRNA) was found in the hypothalamus directly after peripheral injection of IL-1β, where it is able to induce fever [15]. While the concentration in the hypothalamus decreased within 24 h, upregulation of IL-1β mRNA persisted in the cerebral cortex, and this was accompanied by a decrease in spontaneous activity lasting several days. Hypothetically, such persistence of IL-1β transcription might be due to epigenetic changes in microglial cells, a process that is thought to play a role in several neuroinflammatory disorders [29, 30].

Once cytokines have reached the brain, there are changes in behavior through dopamine and serotonin neurotransmitter systems. Cytokines can influence dopamine synthesis via oxidative stress and disruption of the enzyme tetrahydrobiopterin (BH4), which is important for conversion of phenylalanine to the dopamine precursor tyrosine and l-3,4-dihydroxyphenylalanine (l-dopa). In addition, cytokines can enhance dopamine transporter activity and dopamine receptor functioning. Alternatively, cytokines can affect serotonin functioning through the activation of indoleamine 2,3dioxygenase (IDO) in peripheral immune cells or microglia and kynurenine pathways [31–34]. Immunotherapy models have identified dissociation between the role of dopamine and serotonin in symptom expression, with mood and cognitive symptoms being more responsive to treatment with serotonin reuptake inhibitors (SSRIs) and fatigue and psychomotor functioning being more responsive to treatment with dopaminergic medications [35–37]. This suggests that fatigue symptoms may involve alterations in dopamine functioning. Indeed, animal studies show that dopamine depletion alters motivational behavior in a way similar to cytokine administrations [38–41], and it has been demonstrated that immune-induced reductions in physical activity and effort expenditure can be reversed with dopamine treatment [14, 42]. In addition, fatigue is a common symptom in many psychiatric and neurological conditions that have been associated with alterations of the dopamine system including Parkinson’s disease and depression [35, 43–45]. Besides their effects on brain neurotransmitter systems, IL-1 can also influence brain functioning through their effect on hippocampal neuroplasticity and neurogenesis [46] or via neuro-endocrine mechanisms involving the hypothalamic pituitary-adrenal axis (HPA) functioning [47]. These effects have been associated with the development of mental problems that often concur with fatigue symptoms, such as impairments in learning and memory and depressive-like behavior.

To give a clear view of the possible role of IL-1 in the development of fatigue in different diseases, we will discuss the studies that have been performed.

## Overview of studies investigating the role of interleukin-1 in disease

### Inflammatory illnesses

#### Rheumatoid arthritis

Rheumatoid arthritis (RA) is a chronic disease characterized by recurrent, often symmetrical destructive arthritis. In addition to local joint inflammation, RA is known for systemic symptoms such as fatigue. The prevalence of fatigue in the RA population varies between 40 and 88%, depending on criteria and questionnaires used [48–50]. Although the exact causal mechanism of fatigue is unknown [51], it can be predicted by pain, sleep disturbances, and depression, rather than by disease activity [52]. The contribution of cytokine disturbances to the development of fatigue remains to be elucidated but could be prominent as treatment with tumor necrosis factor alpha (TNF-α) inhibitors has a positive effect on fatigue compared to treatment with methotrexate alone [53].

Cytokine disturbances in RA are well known and are predominantly driven by increased TNF-α and IL-1, although TNF-α is measured more frequently. Both concentrations of IL-1β and IL-1Ra are slightly elevated in RA, and both correlate with disease severity, reflected by elevated pain scores and an increased erythrocyte sedimentation rate (ESR) [54, 55]. Several findings suggest a central activation of the immune system in RA patients. A study evaluating IL-1 concentrations in cerebrospinal fluid (CSF) in 14 female RA patients with moderate disease activity and 12 healthy subjects found IL-1β concentrations in CSF are increased in patients and positively correlated to fatigue severity (*R* = 0.55, *p* < 0.05) [26]. Such a correlation was not present for pain or tender joint count. IL-1Ra in CSF was lower in RA patients compared to healthy subjects. Furthermore, IL-1β concentrations in CSF were significantly higher than that in plasma, which suggests a central pro-inflammatory state in RA patients.

The next step is to assess the effect of IL-1 blockade on fatigue severity in RA, which has been investigated by using monoclonal antibodies against IL-1β (canakinumab, Ilaris) and recombinant IL-1Ra (anakinra, Kineret) in patients with current disease activity [1, 2]. In both studies, there was a significant decrease of fatigue severity. The double blind study performed by Alten et al. [1] measured fatigue using the “Functional Assessment of Chronic Illness Fatigue” (FACIT-F) questionnaire in patients on different canakinumab dosing regimens next to methotrexate, compared to patients who used placebo. At 12 weeks, two out of three canakinumab groups reported a small but significant decrease in fatigue compared to placebo. With respect to disease response rate, measured by joint inflammation and other disease-specific characteristics, there was only a significant response in one of the groups (150 mg canakinumab s.c. once every 4 weeks). An inherent problem with canakinumab, being a monoclonal antibody, is its failure to reach the CNS, and hence only fatigue driven by peripherally produced IL-1 that may gain access to the brain is being countered. In case of apparent peripheral inflammation, which is the case in RA, this appears to be effective as can also be concluded from a study lowering TNF-α using a monoclonal antibody; here, a rapid effect on central nociceptive brain activity was found [56].

In the study using anakinra in RA, eight patients were treated daily for 8 weeks, although there was no placebo-treated control group [2]. The decrease of fatigue severity was most profound in the first 4 weeks with visual analog scale (VAS) scores being almost reduced by 50%. Decrease of fatigue was paralleled by a decrease in disease activity.

#### Sjögren’s syndrome

Another disease that is often accompanied by joint pain is Sjögren’s syndrome, although diminished salivary and lacrimal gland function are the hallmarks. Sjögren’s syndrome is characterized by autoantibody production against ribonucleoparticles and mononuclear cell accumulations in exocrine glands. Besides sicca complaints, fatigue is one of the most frequently noted symptoms in this disease reported by up to 85% of patients [57]. Fatigue for some part can be explained by an altered sleeping pattern [58], but IL-1 might also be a contributor.

Harboe et al. assessed IL-1 alterations in CSF in 54 adult patients with primary Sjögren syndrome (pSS) compared to 53 controls [59]. IL-1β concentrations were below the detection limit of 1 pg/ml for both patients and controls. IL-1Ra concentrations were significantly elevated in patients and correlated to fatigue severity using a visual analog scale (VAS) independent of age and depression, although this correlation was very weak (*r* = 0.11, *p* = 0.015).

The effect of IL-1 inhibition on fatigue severity was assessed by the same study group in 26 pSS patients [3]. Patients were treated with either daily anakinra or placebo for a period of 4 weeks and were randomized on a 1:1 basis. Fatigue scores measured with the fatigue severity scale (FSS) after 4 weeks compared to baseline did not differ between groups. However, significantly more patients in the anakinra group had a fatigue reduction of more than 50% when using the VAS fatigue scale (*p* = 0.03). This study suggests anakinra could be effective for treating fatigue in pSS, although the study was probably underpowered to detect significant changes.

#### Cryopyrin-associated periodic syndrome

In cryopyrin-associated periodic syndrome (CAPS), a group of rare diseases with an estimated prevalence of 1 in 360,000 persons [60], increased IL-1β activity plays a crucial role. CAPS consists of three auto-inflammatory disorders: familial cold autoinflammatory syndrome (FCAS), Muckle-Wells syndrome (MWS), and chronic infantile neurologic cutaneous and articular syndrome (CINCA). These syndromes are all caused by a mutation in the NLRP3 gene encoding cryopyrin, a protein which is responsible for inflammasome activation [61]. Different stimuli, for example, cold temperature, in FCAS, can lead to cryopyrin production in these patients, causing a systemic inflammatory response mainly caused by IL-1β. FCAS, MWS, and CINCA are all characterized by intermittent episodes of fever, headache, urticarial rash, and arthralgia [62]. Although these symptoms are typically present during exacerbations, overall quality of life is also significantly affected and fatigue is reported by more than 75% of FCAS patients [63, 64].

The influence of blocking IL-1 on disease severity and fatigue was assessed in several studies. It should be noted that the MWS and CINCA patients tend to have sterile chronic meningitis, which probably results in inhibitors having greater entry into the brain [65]. Koné-Paut et al. assessed the influence of treatment with canakinumab in 35 CAPS patients [66]. At baseline, mean FACIT-F scores for the whole group were 27.4; after 8 weeks of treatment, the score increased to 40.6, which is a significant decrease in fatigue (*p* < 0.05). Symptoms of fatigue, as rated by the physicians, were already absent in more than 85% of patients after 8 days of treatment. In the second part of the study, patients were randomized to either canakinumab or placebo. In those patients randomized to placebo, fatigue recurred. In another study, the influence of canakinumab on fatigue was assessed in seven pediatric CAPS patients [67]. At several time points, physicians scored fatigue severity using a 5-point scale. At baseline, fatigue was reported to be severe in two patients, moderate in three patients, and mild in one patient. After 1 day of treatment, fatigue was absent in five patients and minimal in two patients and this effect was maintained until the next relapse of fever.

The effect of rilonacept (Regeneron), a soluble IL-1 decoy-receptor construct, was assessed in 47 CAPS patients in two sequential phase III studies [68]. In the first double-blind part of the trial, patients were randomized between weekly rilonacept and placebo for a duration of 6 weeks. In the subsequent second study, patients were treated with active drug for 9 weeks, followed by another placebo-controlled period of 9 weeks. Fatigue severity was measured using a 10-point rating scale by both patients and investigators. In both groups, fatigue decreased significantly during the first phase of the trial, with a larger decrease in the rilonacept group. In the third phase of the trial, patients on placebo had a relapse of symptoms, while patients receiving rilonacept remained without fatigue.

The influence of anakinra on the development of symptoms in FCAS was assessed in patients who were exposed to a cold challenge [69]. In three patients, anakinra was given 24 and 1 h prior to the challenge. None of the patients developed acute symptoms which they developed without prior anakinra treatment. Although not measured objectively, patients reported less fatigue and increased well being, a feeling that lasted 48–72 h after the second anakinra dose. In all of the described studies, the decrease in fatigue was accompanied by less inflammatory activity both clinically and biologically. These studies demonstrate the effect of IL-1 on clinical symptoms and the fast improvement of these symptoms when IL-1, especially IL-1β, is inhibited.

#### Sarcoidosis

In sarcoidosis, an inflammatory disease of unknown etiology, patients develop granulomas in involved organs. The lungs are affected most often, but extra-pulmonary manifestations are present in up to 30% of patients [70]. Young patients are most often affected, and symptoms usually resolve within 2–4 years. Even when in clinical remission of the disease, prevalence of fatigue is rather high. In a Dutch post-sarcoidosis cohort of 75 patients, 49% of patients reported severe fatigue, which was associated with psychological distress and reduced health status [71].

To explore the involvement of pro-inflammatory cytokines in post-sarcoidosis patients with fatigue, 72 patients were included in a study by Korenromp et al. [72]. Patients were categorized as being fatigued based on a Checklist Individual Strength subscale fatigue (CIS-f) score ≥35 (*n* = 34) or non-fatigued when the score was below 35 (*n* = 38). Whole blood IL-1α and IL-1β production was measured after lipopolysaccharide (LPS) stimulation. In plasma, these cytokines were also determined in addition to IL-1Ra. No differences for these proteins could be found between groups. The contribution of IL1β was also assessed in 22 patients with active sarcoidosis compared to 22 controls [73]. Fatigue was measured using the Multidimensional Fatigue Inventory (MFI-20), and IL-1β concentrations were determined before and after 11–15 min of cardiopulmonary exercise testing. Between patients and controls, there were no differences measured in IL-1β concentrations. However, pre-exercise circulating IL-1β concentrations in patients significantly correlated with fatigue severity in those patients who used immunomodulatory drugs (*n* = 13). Thus, fatigue in sarcoidosis patients seems to be a consequence of treatment rather than of the disease itself. However, the study population is too small to draw firm conclusions. The effect of IL-1 inhibition on fatigue severity in sarcoidosis patients has not been assessed.

### Non-inflammatory illnesses

#### Diabetes mellitus

During the past three decades, a large number of studies have documented a role of IL-1β in type 1 and type 2 diabetes. IL-1β causes selective pancreatic beta-cell toxicity, resulting in decreased insulin production [74]. Anakinra might be able to reduce this, disease-characterizing, islet inflammation in newly diagnosed type 1 diabetes patients [75] but probably has to be combined with T cell targeting therapy to reach a maximal effect. The effect of anakinra on diabetes regulation was also assessed in type 2 diabetes [76]. After 13 weeks of treatment, patients needed less diabetes lowering drugs to obtain the same glycemic control. A similar positive response on glycemic control was established using an anti-IL-1β antibody in type 2 diabetes [77].

The interaction between peripheral inflammation and deregulation of central mechanisms was demonstrated in type 2 diabetic mice [78]. After administration of LPS or IL-1β, diabetic mice had prolonged sickness behavior compared to controls. The mechanism for this diabetes-induced brain immune alteration is unclear, but it appears that diabetes has an effect on the IL-1β counterregulation, as IL-1Ra did not increase after LPS administration in diabetic mice.

Both patients with type 1 and type 2 diabetes experience fatigue, although literature on this subject is scarce. In a recent study in 214 patients with type 1 diabetes, severe and persistent fatigue was present in 40% of patients [79]. Diabetes appeared to be correlated with behavioral variables rather than with blood glucose concentrations. These results lead to the development of a behavior-based therapy to treat fatigue in type 1 diabetes [80]. Cavelti-Weder et al. assessed the efficacy of XOMA052, a monoclonal anti-IL1β antibody, compared to placebo in 30 type 2 diabetes patients [4]. Fatigue was reported by 53% of patients and significantly correlated to diabetes duration, but not to age, HbA_1c_, weight, body temperature, and C-reactive protein. After treatment for 1 month, fatigue decreased in the groups treated with moderate- and high-dose XOMA052, whereas an increase of fatigue was seen in the low-dose and placebo groups.

#### Cancer

In cancer, fatigue is one of the most prominent symptoms during all stages of disease, leading to substantial impairment and disability. A recent study evaluated the prevalence of fatigue in patients with breast, prostate, colorectal, and lung cancer undergoing active treatment (*n* = 2177) or who had survived cancer (*n* = 515) [81]. Moderate-to-severe fatigue was reported by 45 and 29% of patients, respectively. The impact of fatigue on daily functioning in these patients is even greater than that of nausea or cancer-related pain [82]. The exact mechanism causing fatigue during and after cancer treatment is not clear, but it is suspected that pro-inflammatory cytokines, especially TNF-α and IL-1β play an important role [83]. One of the major reasons for this suspected relationship is that chemotherapeutic agents are known to trigger IL-1β release, as mentioned previously [84]. In the acute situation, such cytokine release promotes survival, but during the course of anti-cancer treatment, it is associated with a variety of manifestations of illness, including fatigue [85]. A systematic review evaluating the relationship between IL-1 and fatigue in different types of cancer during and after treatment could not prove IL-1β concentrations to be significantly correlated to fatigue severity [86]. Patients in different stages of disease were analyzed as one group, which could have influenced the results. It is known that different biological processes take place during treatment and in the post-treatment situation. However, fatigue could be associated with an increase in circulating IL-1Ra (*r* = 0.24, *p* < 0.001) in this review, thus probably pointing to IL-1 activity.

In addition to a possible effect of IL-1 during cancer treatment, IL-1 may also influence the persistence of symptoms after treatment. This was evaluated in a group of advanced cancer patients (*n* = 45) and cancer survivors (*n* = 47) [87]. In both patient groups, IL-1Ra correlated with physical fatigue (*r* = 0.32, *p* = 0.03 and *r* = 0.24, *p* = 0.10, respectively). In cancer survivors, IL-1Ra correlated not only with physical fatigue but also with mental fatigue (*r* = 0.35, *p* = 0.02). When comparing both groups, inflammatory markers were higher in patients with advanced cancer than in cancer survivors. Concentrations of circulating IL-1β and/or IL-1α were not determined.

##### Prostate cancer

A possible relationship between IL-1 and fatigue in patients treated for prostate cancer has already been addressed more than two decades ago [88]. In this study, 15 men undergoing external beam radiation therapy for prostate cancer were evaluated for a period of 8 weeks. Radiation therapy initiates an immunological response to stimulate tissue repair, which is accompanied by an increase in pro-inflammatory cytokines [89]. Patients reported on fatigue daily using a VAS. IL-1β was determined in serum before the start of therapy and weekly thereafter. Both concentrations of IL-1β and fatigue increased during treatment, with a maximum after 4 weeks of treatment. A correlation between these measurements was not determined. Although performed in a small number of patients, this study was the first study on this subject. More recently, other investigators evaluated inflammatory markers during radiation therapy in patients with breast (*n* = 28) and prostate (*n* = 20) cancer [90]. Circulating IL-1β increased significantly during treatment, although there was a large variation between patients, and there was no correlation between IL-1β and fatigue severity. However, in a subset of 22 patients, IL-1Ra was determined, which did correlate with reported fatigue. In another study, a correlation between IL-1β and fatigue was not found [91].

A study conducted in patients with prostate cancer evaluated the influence of single-nucleotide polymorphisms (SNPs), which are associated with the production of pro-inflammatory cytokines. The study assessed the development of fatigue during androgen-deprivation therapy [92]. Testosterone is suspected to modulate cytokine concentrations, especially IL-1β, IL-6, and TNF-α. Variation in IL-1β genotypes did not predict changes in fatigue scores in the 53 patients evaluated. Interventions directed towards inhibition of IL-1 have not been performed in prostate cancer.

##### Breast cancer

Several studies have been performed in breast cancer patients undergoing radio- or chemotherapy. Geinitz and colleagues investigated the association between fatigue and cytokine concentrations during adjuvant radiotherapy in breast cancer patients [93]. In accordance with prostate cancer patients undergoing radiotherapy, fatigue severity reached a maximum after 4 weeks of treatment; IL-1β concentrations in serum did not change and did not correlate with fatigue severity. Another study examined potential predictors of fatigue before, during, and after adjuvant therapy in 44 women after breast cancer surgery [94]. Blood samples were collected before adjuvant therapy had started. Questionnaires were repeated during and after therapy. Before adjuvant therapy, higher IL-1β concentrations predicted fatigue severity. During and after adjuvant therapy, this association was no longer present, but cytokine concentrations were not determined during this period. Liu et al. measured fatigue and IL-1Ra in a group of 53 women diagnosed with breast cancer before and during chemotherapy [95]. At baseline, IL-1Ra did not correlate with higher fatigue levels and had no influence on changes of fatigue severity during treatment. The most recent study, performed by Schmidt et al., did find a small but significant influence of increased IL-6/IL-1Ra ratio after treatment, which could not be found for IL-1Ra levels (*r* = 0.25) [96].

Besides experiencing fatigue during cancer treatment, breast cancer survivors up to 2 years after completing treatment also report more fatigue than healthy controls [97]. This symptom may be due to the cytokine response initiated by tissue damage during the acute treatment phase and persists after several years. To investigate the contribution of pro-inflammatory cytokines to fatigue after treatment, Bower et al. compared 20 fatigued women with 20 women without fatigue between 1 and 5 years after breast cancer diagnosis [98]. Fatigued women had significantly higher concentrations of IL-1Ra in serum (*p* = 0.006); there were no differences for IL-1β concentrations, which were below the detection limit in almost half of the patients. These observations were not confirmed in a study in 103 patients 1–3 months after treatment for breast cancer [99]. Bower et al. also evaluated ex vivo whole blood IL-1β production after LPS stimulation in 10 fatigued and 15 non-fatigued breast cancer survivors at baseline and after completion of the Trier Social Stress Test (TSST) [100]. At baseline, there were no differences with regard to IL-1β production. However, after completing the TSST, fatigued patients had significant higher IL-1β concentrations. These findings suggest a higher pro-inflammatory response to psychological stress in fatigued patients. Circulating IL-1Ra concentrations were determined by the same study group in 50 fatigued and non-fatigue breast cancer survivors and were found to be significantly higher in fatigued patients [101]. Again, this finding was contradicted by a cross-sectional study evaluating IL-1Ra levels in 299 disease-free breast cancer survivors, who did not find any positive correlations between this marker and fatigue severity [102].

The presence of SNPs in promoters of cytokine genes was also studied in breast cancer survivors (fatigued *n* = 33, non-fatigued *n* = 14). The presence of at least one cytosine nucleotide at the IL-1β gene (rs16944), a common SNP in many diseases, was reported to be associated with fatigue [103]. However, in a larger cohort (*n* = 302), this association could not be confirmed [104].

##### Other types of cancer

In two other types of solid tumors, the involvement of IL-1 in the development of fatigue has been assessed. Orre et al. evaluated 92 fatigued testicular cancer survivors, compared to 191 non-fatigued survivors at a median of 11 years after diagnosis [105]. Cases had significant higher IL-1Ra concentrations than controls. Increased IL-1Ra concentrations significantly correlated with physical fatigue, although they explained only 4% of variance in logistic regression analysis. A study investigating IL-1 in 15 patients with uterine cancer before, during, and after undergoing curative radiation therapy failed to prove a correlation, as IL-1 concentrations remained below the detection limit during the whole study [106]. No distinction was made between IL-α and IL-1β in this small pilot study.

In hematologic malignancies, a single study has been performed that assessed the correlation between fatigue and IL-1 and IL-1Ra in 54 patients with acute myeloid leukemia or myelodysplastic syndrome undergoing pretreatment evaluation [107]. IL-1Ra concentrations correlated with fatigue severity (*r* = 0.52). Concentrations of circulating cytokines were higher in patients than in healthy controls.

The effect of IL-1α inhibition, using a neutralizing antibody, on fatigue was determined in 16 patients with metastatic, treatment-resistant non-small cell lung cancer [108]. Quality of life was assessed at baseline and after 8 weeks of treatment using the European Organization for Research and Treatment of Cancer, Quality of Life Questionnaire (EORTC QLQ C-30). After 8 weeks, fatigue was reported to be less severe, although this difference was not significant probably due to the small patient numbers. A significant improvement of fatigue after blocking IL-1α was seen in a large group of patients treated for metastatic colorectal cancer, in addition to improvement of appetite and a decrease in pain severity (personal communications) [109].

#### Post-stroke fatigue

In patients who experienced a stroke, fatigue is reported by 29–77% of the population. The prevalence of fatigue is equally distributed over patients after ischemic stroke and those who had an intracerebral hemorrhage [110]. With respect to inflammation, high levels of circulating IL-6 during the acute phase of stroke have been associated with poor outcome (odds ratio 3.1, 95% CI 1.9–5.0); these data are derived from a large prospective study consisting of 844 patients [111]. In subarachnoid hemorrhage patients, IL-6 concentrations can be lowered using intravenous anakinra infusion [112] and might prove to increase survival in future studies.

The relationship between post-stroke fatigue and inflammation was described by Ormstad et al., who included 45 patients after a first stroke in a longitudinal study [113]. Serum samples were collected <24, 24–48, and 48–72 h after stroke onset in 35, 7, and 3 of the 45 patients. IL-1β and IL-1Ra were measured in available samples. Fatigue was measured using the Fatigue Severity Scale (FSS) up to 18 months after stroke. Directly after stroke, IL-1β concentrations correlated with fatigue severity after 6 months (*r* = 0.37, *p* = 0.015); this correlation could no longer be found after 12 and 18 months. At 12 months, however, a negative correlation between IL-1Ra in the acute phase and fatigue was found (*r* = −0.38, *p* = 0.013), a correlation that was not present at 6 and 18 months. Age, gender, comorbidity, and the use of medication were not confounders for these associations. These results imply that the acute inflammatory response during stroke has an impact on the occurrence of fatigue in the chronic phase.

In a study of 39 stroke patients, the presence of a C allele at a SNP located in the promoter region of *IL1RN* was related to the severity of post-stroke fatigue [114]. The presence of a C allele in this region has been associated with lower IL-1Ra concentrations and higher concentrations of circulating IL-1β [115]. In this study, patients were included within 72 h of stroke onset; fatigue was assessed using the Fatigue Assessment Scale (FAS) at one or more time points (30–365 days after stroke). In patients with severe fatigue, a C/T or C/C genotype was significantly more present (88%) than in patients with moderate (57%) and low fatigue (24%, *p* = 0.03). This small study is the only study performed in this field, and circulating cytokine concentrations were not determined.

#### Chronic fatigue syndrome

Chronic fatigue syndrome (CFS) is a condition of unknown origin that is characterized by the presence of severe fatigue for a duration of at least 6 months, next to several accompanying symptoms such as headaches, sore throat, and muscle and joint pain [116]. Over the past decades, CFS has been attributed to a range of different causes, but a unifying cause has not been found. Even if a distinct abnormality is found repeatedly, for example relative hypocortisolism [117], it is difficult to determine whether this is a causative factor or rather an epiphenomenon as a consequence of inactivity, depressive symptoms, sleep problems, etc. Perhaps, more than any other chronic disease associated with fatigue, cytokines have been measured by several investigators. A relationship between IL-1 and fatigue severity has often been assessed. The studies reveal a large heterogeneity, not only with respect to patient characteristics but also with respect to selection of controls and sample handling. In addition, there is a large variation in questionnaires used to measure fatigue dimensions and fatigue-related symptoms. These issues make it difficult to draw reliable conclusions.

A systematic review focusing on circulating cytokines in CFS was published recently by Blundell et al. [118], who reviewed all studies published on this subject between the publication of the first CFS case definition in 1988 [119] to March 2015. All 38 studies measuring circulating cytokines in diagnosed CFS patients compared to controls were included. As mentioned earlier, there were large differences with respect to recruitment of controls, sample handling, and exclusion of concomitant diagnoses. IL-1α was measured in 11 of the described studies, 27% of studies found increased concentrations, and 73% found no significant differences. IL-1β was determined in 28 studies, with only 25% reporting increased concentrations; the other studies did not find any significant differences. One of the more recent studies included in the review also discriminated patients with a short duration of illness (≤3 years, *n* = 52) from patients with a long illness duration (*n* = 246) and controls (*n* = 348) [120]. It appeared that patients with a short duration of illness had significantly higher IL-1α and IL-1Ra concentrations than controls. This was also found when comparing IL-1β levels in patients with short versus long illness duration. IL-1β appeared to be elevated in patients with a short duration and decreased in patients with a long illness duration (when compared to controls). After this extensive review of the literature by Blundell, three more studies on circulating cytokines were published [121–123]. A study by Russell et al. also tried to discriminate between patients with different illness durations [123]. Comparing IL-1 concentrations, no differences could be found, although it has to be noted that patients with a “short” illness duration had been fatigued for a mean of 7 years, which is longer than the study mentioned earlier. In the linear classification model, however, IL-1α appeared to have predictive value in recently ill adolescent patients. Hardcastle et al. compared severely ill, house-bound patients (*n* = 19), to moderately ill patients (*n* = 22) [121]. Although groups are rather small, IL-1β was significantly elevated in the moderately ill patients (*p* = 0.002). There were no differences for IL-1Ra. The third study could not find any differences between patients and controls for either IL-1β or IL-1α in a group of 100 patients and 79 controls [122]. We conclude from the literature that there is limited evidence for increased circulating IL-1 in CFS patients, although there might be a more pro-inflammatory pattern in those with a short illness duration [120].

The effect of physical exercise on circulating cytokines was discussed in a separate systematic review [124], although some of the studies discussed were also included in the review by Blundell [125–129]. The conclusion of this review is that also after exercise of varying intensity, there are no consistent differences with respect to IL-1β.

Another approach is to compare cytokine production capacity of PBMCs after stimulation between CFS patients and controls. An early study reported increased IL-1β production after LPS stimulation in a small group of CFS patients (*n* = 9) compared to controls (laboratory personal, *n* = 7) [130]. Swanink et al. recruited neighborhood controls and found the opposite: lower LPS-induced IL-1β concentrations in patients (*n* = 76) than in controls (*n* = 69), with a large overlap between concentrations of cytokines [131]. Lower IL-1β and IL-1α production after PHA stimulation was also reported by Mawle et al. in patients with a gradual onset of symptoms; no differences were observed when those with a gradual and acute onset analyzed together [132]. A fourth study by Cannon et al., published in the same period, investigated IL-1β production in women during different phases of the menstrual cycle [133]. In controls, spontaneous IL-1β production by PBMCs increased during the luteal phase, which already has been observed in healthy subjects many years ago [134]. However, this could not be found in CFS patients. One recent study reported no differences between CFS patients and controls [135].

IL-1β production by PBMCs in relation to fatigue has also been studied during the acute phase of an infection [136] and in the phase of persisting symptoms [137]. During the acute phase, the IL-1β concentration correlated significantly with fatigue symptoms; however, this relationship disappeared in the persistent phase. The perpetuation of fatigue symptoms in the absence of peripherally increased cytokine concentrations suggest that other, most likely central mechanisms, may be involved in persistent fatigue after an acute infection.

With the brain as the suspected target organ for immunological dysregulation in CFS, a limited number of studies measured cytokine concentrations in cerebrospinal (CSF) fluid of patients. The first study, performed in 1991 by Lloyd et al., found no differences in IL-1β concentrations between patients and controls [138]. Others had similar findings, and both IL-1α and IL-1β tended to be below the detection limit [139, 140]. A more recent study compared 32 CFS patients to 40 patients with multiple sclerosis (MS) and 19 controls [141]. CFS patients had lower CSF concentrations of both IL-1β and IL-1Ra compared to the MS and control group. When CFS patients were compared with MS patients only, IL-1α levels were also significantly lower.

Instead of creating more insight into pathological mechanisms in CFS, the described studies tend to raise more questions with respect to the role of IL-1 in CFS. It could be that disturbances of IL-1 signaling are only present in certain groups, for example only in those patients with short illness duration or those who experience fatigue after an infection, instead of when all patients are considered together. One possible way to elucidate the role of IL-1 in CFS is to investigate the effect of blocking IL-1 on fatigue severity in CFS patients [142].

## Conclusions

In this review, we first described the mechanism by which IL-1 is able the influence certain brain regions, thereby leading to the development of fatigue. Next, we reviewed the literature describing studies where (i) fatigue was correlated to IL-α, IL-1β, or IL-1Ra activity or (ii) the effect of lowering IL-1 concentrations on fatigue severity was measured. In addition to inflammatory diseases such as CAPS, we also focused on non-inflammatory diseases characterized by profound fatigue, such as several malignancies and CFS. There might be a distinctive underlying mechanism causing fatigue in inflammatory disorders, compared to the other groups of fatigue causing illnesses. In inflammatory diseases, fatigue often has an acute pattern; however, in subgroups of patients, fatigue persists even when the inflammation phase has subsided.

It can be concluded that there is no solid evidence that increased concentrations of circulating IL-α and IL-1β are associated with fatigue in any of the diseases described. This is not surprising, given the fact that circulating concentrations of these cytokines usually are very low, as discussed previously [12]. However, IL-1Ra seems to be correlated with fatigue in some diseases, for example in cancer. However, in each of the studies described in this review, but especially in CFS, studies are rather contradicting. For a large part, this can be caused by the fact that there is a large heterogeneity between studies. Selection of controls and sample handling, which is known to be very important when measuring cytokines, differed significantly between studies or was not described [143]. Furthermore, studies differed with respect to questionnaires used to measure fatigue, the presence of comorbid diseases, the use of medication in the patients studied, sample size, time since onset of the disease, duration of the fatigue (acute versus chronic), and the presence or absence of inflammatory processes.

For blocking IL-1 activity, most of the currently available inhibitors do not reach effective concentrations in the brain when the blood brain barrier is intact. This particularly is the case for the large molecular inhibitors (like canakinumab and rilonacept). For anakinra, which has a smaller molecular weight of 17 kDa, the available pharmacological data show that the drug is able to reach the CNS after peripheral administration, although it is not clear if the local concentration in the CNS is high enough to have a substantial influence on neural processes [144, 145].

In diseases such as rheumatoid arthritis [2] and Sjögren’s syndrome [3], blocking IL-1 using anakinra reveals promising effects on fatigue. In addition, specific inhibition of either IL-1α [109] or IL-1β [4] also has a positive influence on fatigue severity. Unfortunately, the majority of the studies were not randomized controlled trials [2, 4] or were most likely underpowered to detect significant effects [3]. If IL-1 blockade effectively diminishes fatigue, the question of course remains whether this is a direct effect on central fatigue, whether the effect on fatigue is due to inhibition of inflammation, or whether IL-1 blockade directly affects central neurotransmitter systems. Also, it is important to determine if the positive effects of IL-1 blockade are limited to acute fatigue or are also present in patients who report persistent fatigue without evidence of being ill. Especially in this last group, persistent fatigue may involve maintenance of alterations in central brain systems, potentially triggered by acute inflammation.

With regard to future studies, it is our hope that these will be performed in more controlled settings, which will make it easier to draw conclusions and to establish whether fatigue should or should not be added to the growing list of diseases in which blocking IL-1 is effective [146].
